# Identification and Characterization of a Translational Mouse Model for Blood–Brain Barrier Leakage in Cerebral Small Vessel Disease

**DOI:** 10.3390/ijms26146706

**Published:** 2025-07-12

**Authors:** Ruxue Jia, Gemma Solé-Guardia, Vivienne Verweij, Jessica M. Snabel, Bram Geenen, Anil Man Tuladhar, Robert Kleemann, Amanda J. Kiliaan, Maximilian Wiesmann

**Affiliations:** 1 Department of Medical Imaging, Anatomy, Radboud University Medical Center, Donders Institute for Brain, Cognition & Behavior, Center for Medical Neuroscience, Preclinical Imaging Center PRIME, Radboud Alzheimer Center, Radboudumc, 6525 EZ Nijmegen, The Netherlands; ruxue.jia@radboudumc.nl (R.J.); gemma.soleguardia@radboudumc.nl (G.S.-G.); vivienne.verweij@radboudumc.nl (V.V.); bram.geenen@radboudumc.nl (B.G.);; 2 Department of Neurology, Research Institute for Medical Innovation, Donders Institute for Brain, Cognition and Behavior, Radboudumc, 6525 GA Nijmegen, The Netherlands; anil.tuladhar@radboudumc.nl; 3 Department of Metabolic Health Research, The Netherlands Organization for Applied Scientific Research (TNO), 2333 CK Leiden, The Netherlandsrobert.kleemann@tno.nl (R.K.)

**Keywords:** blood–brain barrier dysfunction, cerebral small vessel disease (cSVD), dynamic contrast-enhanced MRI (DCE-MRI), endothelial dysfunction, neuroinflammation

## Abstract

Blood–brain barrier (BBB) dysfunction is a hallmark of cerebral small vessel disease (cSVD). This study aimed to identify a mouse model that replicates BBB impairment and shares key cSVD risk factors. Transgenic db/db and LDLr^−/−^.Leiden mice, both prone to obesity and hypertension, were compared to C57BL/6J controls. BBB leakage was assessed using DCE-MRI and sodium fluorescein (NaFl); cerebral blood flow (CBF) by MRI. Dyslipidemia and vascular inflammation were measured by plasma tests. Tight junction integrity, endothelial dysfunction (glucose transporter 1, GLUT-1) and neuroinflammation were evaluated with immunohistochemistry and PCR. Both transgenic models developed an obese phenotype with hyperinsulinemia, but only LDLr^−/−^.Leiden mice showed human-like dyslipidemia. When fed a high-fat diet (HFD) or HFD plus cholesterol, LDLr^−/−^.Leiden mice showed reduced CBF, endothelial dysfunction (lowered GLUT-1), elevated vascular inflammation (ICAM-1, VCAM-1, S-selectin), and BBB leakage, as evidenced by DCE-MRI and NaFl, together with reduced ZO-1 and claudin-5 expression. Contrastingly, db/db mice showed endothelial dysfunction without BBB leakage. Neuroinflammation (IBA-1, GFAP) was observed only in LDLr^−/−^.Leiden groups, consistent with BBB disruption. These findings indicate that LDLr^−/−^.Leiden mice, but not db/db mice, are a promising translational model for studying BBB dysfunction in cSVD, offering insights into disease mechanisms and a platform for therapeutic development.

## 1. Introduction

Cerebral small vessel disease (cSVD) refers to a group of heterogeneous disorders characterized by pathological processes associated with the cerebral microvasculature [1,2]. CSVD affects nearly all individuals over 60 years of age and is a significant contributor to stroke, cognitive decline, dementia, and physical disabilities [1,3]. Due to heterogeneous clinical manifestations and the absence of evident symptoms at an early stage, imaging techniques such as magnetic resonance imaging (MRI) are commonly used to support diagnosis in clinical practice [4,5]. Characteristic MRI features such as white matter hyperintensities (WMH), lacunes, brain atrophy and reduced cerebral blood flow (CBF) are important in assessing cSVD severity and prognosis [4,5]. Despite the increasing awareness of cSVD as a prevalent and important pathological neurological disease among the aging population, the exact pathophysiological mechanisms of cSVD remain incompletely understood.

Endothelial dysfunction, blood–brain barrier (BBB) dysfunction and neuroinflammation are considered critical mechanisms and pathological changes contributing to the onset and progression of cSVD [6]. Brain microvascular endothelial cells (ECs), together with pericytes and astrocytes, participate in the formation and maintenance of the BBB, which protects the brain by preventing harmful agents from entering the brain [7,8]. Very dense tight junctions (TJs) between ECs and extraordinarily low transcytosis rates in brain microvascular ECs play crucial roles in sustaining the BBB integrity [9]. Moreover, the BBB can regulate CBF through the intrinsic properties of ECs and their interactions with neurons, ensuring proper brain function. Some studies have demonstrated that cSVD risk factors such as hypertension, hyperlipidemia, and obesity can impair BBB integrity by inducing endothelial dysfunction and TJ disruption through multiple pathways such as inflammation and oxidative stress [10,11,12]. BBB leakage not only allows peripheral inflammatory factors and blood components to enter the perivascular space, further damaging TJs and aggravating BBB damage [13], but it also activates various signal cascades in microcirculatory ECs [1]. This leads to the release of adhesion molecules, including intercellular adhesion molecule 1 (ICAM-1) and vascular cell adhesion molecule 1 (VCAM-1), which further aggravate local inflammation and lead to endothelial and vascular dysfunction. Additionally, these adhesion molecules facilitate the migration of circulating leukocytes into brain tissue, intensifying neuroinflammation [1,11]. This vicious cycle ultimately contributes to neuronal damage and cognitive decline [1]. Therefore, understanding the mechanisms underlying BBB leakage in cSVD is crucial to support the development of effective therapeutic strategies.

Given the importance and structural complexity of the BBB, development of highly translational animal models to study BBB leakage is of utmost importance. Translational models should mimic the development, pathophysiological characteristics and risk factors of human cSVD and accurately reflect the pathological changes in the BBB. In addition, some innovative translational research methods currently used to visualize BBB leakage in human research, such as dynamic contrast-enhanced MRI (DCE-MRI), should be applicable to such animal models [14]. Although there are mouse models available to study BBB impairment, such as endothelial-related gene knockout mice or mechanically damaged BBB mouse models [15,16], most of these models can only be used to study BBB changes under acute and very specific experimental conditions and do not show a gradual disease development with risk factors that replicate the clinical presentation of cSVD. Furthermore, these mouse models use invasive methods like Evans blue staining, which are not applicable to human studies [15], thus hampering translational measurements. In short, there are no validated models available yet that reflect BBB leakage and exhibit risk factors of human cSVD, providing readouts for clinical translation.

This study aimed to identify and characterize a suitable translational mouse model that meets the above-described requirements and, secondly, to investigate mechanisms underlying cSVD with particular emphasis on BBB leakage, endothelial and vascular damage and neuroinflammation. Considering vascular risk factors of cSVD such as obesity, hyperglycemia, insulin resistance, and hypertension, two mouse strains were selected. The transgenic leptin receptor-deficient (db/db) mouse is the most widely used mouse model for studying type 2 diabetes, which spontaneously exhibits obesity, hyperglycemia and vascular disorders on a chow diet [17,18,19]. Due to severe metabolic complications and potentially higher mortality, db/db mice are less suitable for long-term studies [20,21]. We also investigated the low-density lipoprotein receptor knockout substrain Leiden (LDLr^−/−^.Leiden) mouse. LDLr^−/−^.Leiden on a high-fat diet (HFD) develops risk factors such as hypertension and dyslipidemia [22]. The LDLr^−/−^.Leiden on HFD with 1% cholesterol (HFD+C) additionally exhibits hypercholesterolemia, which is also an important risk factor for cSVD [20,23]. Among these groups, we hypothesize that a 36-week-old LDLr^−/−^.Leiden mice group fed with HFD may better recapitulate BBB changes under midlife obesity. We used DCE-MRI, arterial spin labeling (ASL), immunohistochemical and other biochemical methods to evaluate BBB integrity, brain and vascular pathology and (neuro)inflammation in both mouse strains as compared to wildtype C57BL/6J (WT), which is the prevailing genetic background strain of both strains. This study will identify and characterize a suitable experimental model, which can be used in the future to investigate underlying processes in cSVD and to develop preventive or therapeutic treatments.

## 2. Results

**Figure 1:**
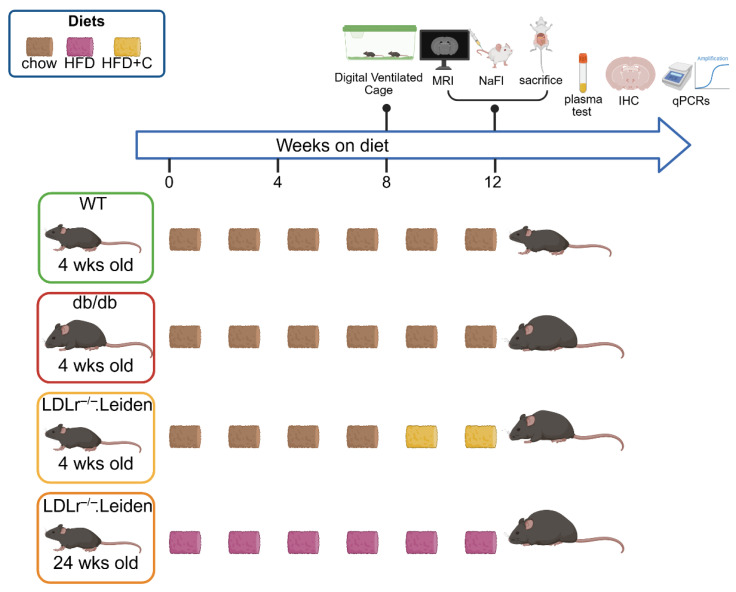
Study design. Mice were housed in groups in digital ventilated cages (DVC) at arrival at 12 weeks and 32 weeks. Mice on the same diet and of the same strain were randomly assigned to groups and housed in separate cages. All mice were housed under identical conditions with ad libitum access to food and water. The mice of group 1 (WT) and group 2 (db/db) were fed a standard diet (chow) throughout the study. Group 3 (younger LDLr^−/−^.Leiden mice) were fed chow from birth to 12 weeks old and switched to a high-fat diet enriched with 1% cholesterol (HFD+C) until 16 weeks old. Group 4 (older LDLr^−/−^.Leiden mice) was fed a high-fat diet (HFD) for 12 weeks starting from 24 weeks of age. Thereafter the mice underwent MRI scanning and were then sacrificed for postmortem studies. Abbreviations: high-fat diet (HFD), high-fat diet with 1% cholesterol (HFD+C), magnetic resonance imaging (MRI), sodium fluorescein (NaFl), immunohistochemistry (IHC), quantitative PCR (qPCRs). The figure was created in BioRender. Jia, R. (2025).

The complete study design is displayed in Figure 1.

### 2.1. Body Weight, Plasma Lipid Changes and Home-Cage Activity

**Figure 2:**
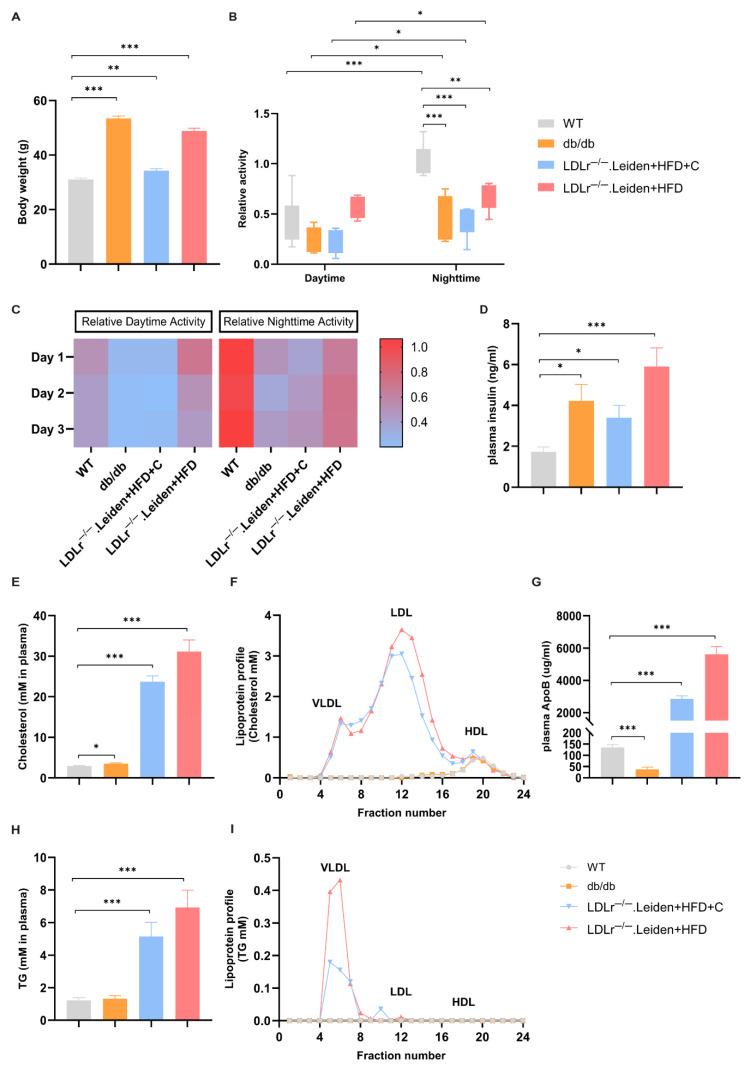
Body weight, plasma lipid changes and home-cage activity in different groups. (**A**) Body weight (group size: WT: *n* = 10; db/db: *n* = 11; LDLr^−/−^. Leiden+HFD+C: *n* = 12; LDLr^−/−^. Leiden+HFD: *n* = 12). (**B**) Relative home-cage activity during the daytime and nighttime was measured in all groups across three days before the MRI scan (group size: WT: *n* = 8; db/db: *n* = 4; LDLr^−/−^. Leiden+HFD+C: *n* = 5; LDLr^−/−^. Leiden+HFD: *n* = 5). (**C**) Heatmaps show the average home-cage activity per day in all groups recorded during the three days before the MRI scan. (**D**) Plasma insulin was determined by ELISA (group size: WT: *n* = 11; db/db: *n* = 11; LDLr^−/−^. Leiden+HFD+C: *n* = 12; LDLr^−/−^. Leiden+HFD: *n* = 12). (**E**) Plasma total cholesterol and lipoprotein profile, and (**G**) plasma ApoB concentration and (**H**) plasma triglyceride (TG) concentration of all groups were determined by enzymatic assays (group size: WT: *n* = 11; db/db: *n* = 11; LDLr^−/−^. Leiden+HFD+C: *n* = 12; LDLr^−/−^. Leiden+HFD: *n* = 12). Lipoprotein profiles were analyzed in plasma pools; in the respective fractions (**F**), cholesterol and (**I**) TG concentrations were determined and plotted as profiles. Data were presented as mean ± SEM. Box plot with 95% confidence intervals for the mean. * *p* < 0.05, ** *p* < 0.01, *** *p* < 0.001.

All transgenic mice were heavier than the WT reference mice, but the LDLr^−/−^.Leiden mice on HFD+C had the least weight gain of these experimental groups (Figure 2A, db/db: *p* < 0.001; LDLr^−/−^.Leiden +HFD+C: *p* = 0.002; LDLr^−/−^.Leiden+HFD: *p* < 0.001).

The activity inside the DVC was determined across three days before the MRI (Figure 2B,C). Being nocturnal animals, all groups of mice were more active during nighttime compared to daytime. There was no significant difference in daytime activity across all groups. Furthermore, nighttime activity was significantly lower in all experimental mouse strains compared to WT mice (Figure 2B, db/db: *p* < 0.001; LDLr^−/−^.Leiden+HFD+C: *p* < 0.001; LDLr^−/−^.Leiden+HFD: *p* = 0.002).

Compared to WT mice, both db/db and LDLr^−/−^.Leiden mice showed higher plasma insulin concentrations (Figure 2D, db/db: *p* = 0.015; LDLr^−/−^.Leiden+HFD+C: *p* = 0.015; LDLr^−/−^.Leiden+HFD: *p* < 0.001). Plasma test results also showed that plasma total cholesterol, ApoB (reflecting the number of VLDL and LDL particles) and triglyceride (TG) concentration of both groups of LDLr^−/−^.Leiden mice were significantly higher than those of WT mice whose plasma lipids were within a normal range (Figure 2E,G,H: LDLr^−/−^.Leiden+HFD+C: *p* < 0.001; LDLr^−/−^.Leiden+HFD: *p* < 0.001). While db/db mice had slightly higher plasma total cholesterol levels than WT mice (Figure 2E, *p* = 0.043), their plasma ApoB concentration was lower than that of WT mice (Figure 2G, *p* < 0.001). Importantly, lipoprotein profile analysis demonstrated that, only in LDLr^−/−^.Leiden mice, circulating cholesterol and TG were predominantly confined to VLDL/LDL lipoprotein particles (Figure 2F,I). By contrast, in WT and db/db mice, plasma cholesterol was confined to HDL particles, which usually served as vascular protective particles (Figure 2F).

### 2.2. Changes in Cerebral Vascular Function and Brain Atrophy

#### 2.2.1. CBF and Brain Atrophy

**Figure 3:**
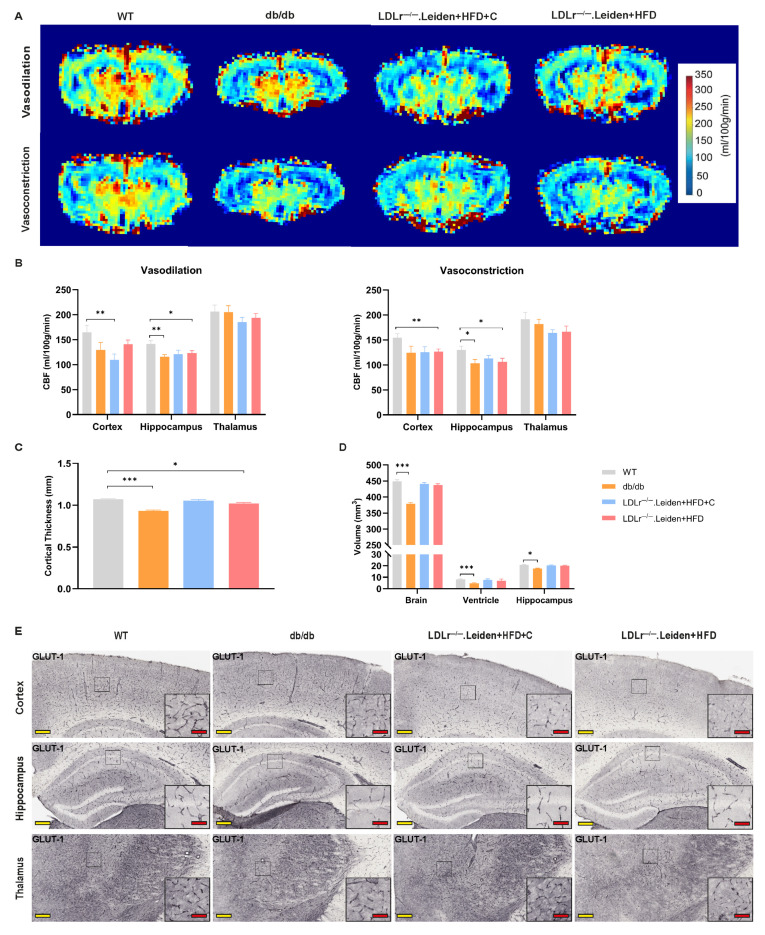

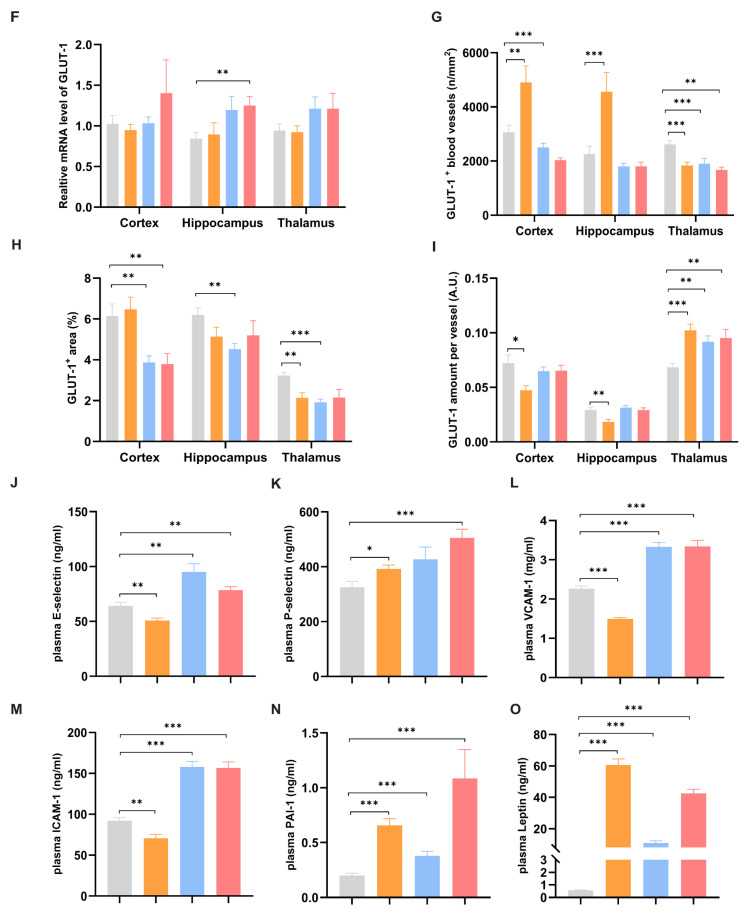
Vascular conditions and brain structure changes. (**A**) Representative high-resolution voxel-wise images of the bregma (−1.94) and (**B**) quantification of cerebral blood flow (CBF) for each mice group under vasodilation condition and vasoconstriction condition (group size: WT: *n* = 10; db/db: *n* = 9; LDLr^−/−^.Leiden+HFD+C: *n* = 12; LDLr^−/−^.Leiden+HFD: *n* = 12). (**C**) Cortical thickness was measured in different groups (group size: WT: *n* = 11; db/db: *n* = 10; LDLr^−/−^.Leiden+HFD+C: *n* = 12; LDLr^−/−^.Leiden+HFD: *n* = 11). (**D**) Brain volumes and ventricular volumes were calculated with FMRIB Software Library (version 5.0.10) based on threshold. Hippocampal volumes were measured on 6 consecutive sections of hippocampus (bregma −1.34 to −3.40) (group size: WT: *n* = 11; db/db: *n* = 11; LDLr^−/−^.Leiden+HFD+C: *n* = 12; LDLr^−/−^.Leiden+HFD: *n* = 12). Immunohistochemical staining for Glucose Transporter 1 (GLUT-1) was performed on the left hemisphere of mice. (**E**) Representative images of GLUT-1 staining in different ROIs (yellow scale bar = 400 µm; red scale bar = 50 µm). (**F**) GLUT-1 gene expression measured by qPCR (group size: WT: *n* = 11; db/db: *n* = 10; LDLr^−/−^.Leiden+HFD+C: *n* = 12; LDLr^−/−^.Leiden+HFD: *n* = 11). (**G**) The number of GLUT-1 positive blood vessels per mm^2^ and (**H**) the percentage of GLUT-1 positive staining per area were representative of capillary density (Group size: WT: *n* = 11; db/db: *n* = 11; LDLr^−/−^.Leiden+HFD+C: *n* = 10; LDLr^−/−^.Leiden+HFD: *n* = 11). (**I**) GLUT-1 amount per vessel represented GLUT-1 transporter density per vessel (group size: WT: *n* = 11; db/db: *n* = 11; LDLr^−/−^.Leiden+HFD+C: *n* = 10; LDLr^−/−^.Leiden+HFD: *n* = 11). Plasma concentration of (**J**) E-selectin, (**K**) P-selectin, (**L**) vascular cell adhesion molecule-1 (VCAM-1), (**M**) intercellular adhesion molecule-1 (ICAM-1), (**N**) plasminogen activator inhibitor-1 (PAI-1) and (**O**) leptin measured with ELISA (group size: WT: *n* = 11; db/db: *n* = 11; LDLr^−/−^.Leiden+HFD+C: *n* = 12; LDLr^−/−^.Leiden+HFD: *n* = 12). Data were presented as mean ± SEM. * *p* < 0.05, ** *p* < 0.01, *** *p* < 0.001.

Vasodilation and vasoconstriction were analyzed by measuring the changes in CBF under a normal gas mix (air/oxygen = 2:1) and pure medical oxygen. As shown in Figure 3A, under vasodilation conditions, cortical CBF in LDLr^−/−^.Leiden mice on HFD+C was lower compared to WT mice (*p* = 0.006). In the hippocampus, db/db mice and LDLr^−/−^.Leiden mice fed a high-fat diet also exhibited lower CBF (Figure 3A,B, db/db: *p* = 0.008; LDLr^−/−^.Leiden+HFD: *p* = 0.042). Under vasoconstrictive conditions, LDLr^−/−^.Leiden mice on HFD showed a significantly lower cortical CBF compared to the WT group (Figure 3A,B, *p* = 0.006), whereas LDLr^−/−^.Leiden on HFD+C mice showed no significant changes. Hippocampal CBF in db/db mice and LDLr^−/−^.Leiden+HFD mice was still significantly lower than that of WT mice (Figure 3A,B, db/db: *p* = 0.024; LDLr^−/−^.Leiden+HFD: *p* = 0.031). No significant difference between WT mice and other experimental groups was found when investigating thalamic CBF (Figure 3A,B).

Ultrahigh field MRI (11.7 Tesla Bruker scanner) was used to visualize cerebral structure and function in vivo. Compared to WT mice, both db/db and HFD-fed LDLr^−/−^.Leiden mice showed decreased cortical thickness (Figure 3C, db/db: *p* < 0.001; LDLr^−/−^.Leiden+HFD: *p* = 0.012). Consistent with the results of cortical thickness, brain volume, ventricular volume and hippocampal volume of db/db mice were all smaller compared to WT mice (Figure 3D, brain: *p* < 0.001; ventricle: *p* < 0.001; hippocampus: *p* = 0.027).

#### 2.2.2. GLUT-1

GLUT-1 is exclusively expressed in brain microvascular ECs, transporting glucose from blood into the brain tissue [24]. GLUT-1 mRNA expression and GLUT-1 immunohistochemistry (IHC) were performed to investigate capillary condition. A higher GLUT-1 mRNA expression was found in the hippocampus of LDLr^−/−^.Leiden mice on HFD compared to those in WT mice (Figure 3F, *p* = 0.005).

GLUT-1 positive blood vessels and GLUT-1 relative positive area were quantified to assess capillary density. The capillary density in both the cortex (Figure 3G, *p* = 0.002) and hippocampus (Figure 3G, *p* = 0.007) of db/db mice was increased compared to WT mice, while the capillary density was lower in the cortex of LDLr^−/−^.Leiden mice fed with HFD+C (Figure 3G, *p* < 0.001). Fewer GLUT-1 positive capillaries were observed in the thalamus of all three experimental groups compared to the WT group (Figure 3G, db/db: *p* < 0.001; LDLr^−/−^.Leiden+HFD+C: *p* < 0.001; LDLr^−/−^.Leiden+HFD: *p* = 0.007). Similarly, db/db mice and LDLr^−/−^.Leiden+HFD mice had a smaller GLUT-1 positive area in the thalamus (Figure 3H, *p* = 0.002) and cortex (Figure 3H, *p* = 0.008), respectively. Likewise, the GLUT-1 positive area was smaller in all brain subregions of LDLr^−/−^.Leiden mice fed with HFD+C (Figure 3H, db/db: *p* = 0.003; LDLr^−/−^.Leiden+HFD+C: *p* < 0.001; LDLr^−/−^.Leiden+HFD: *p* = 0.001).

We further investigated the distribution of GLUT-1 on blood vessels. The presence of GLUT-1 transporters on the cortical and hippocampal vessels of db/db mice was less than that in WT mice, but the presence in the thalamic vessels was significantly increased (Figure 3I, cortex: *p* = 0.01; hippocampus: *p* = 0.007; thalamus: *p* < 0.001). For both groups of LDLr^−/−^.Leiden mice, a significantly larger GLUT-1 amount was only found in thalamic vessels (Figure 3I, LDLr^−/−^.Leiden+HFD +C: *p* = 0.001; LDLr^−/−^.Leiden+HFD: *p* = 0.005).

#### 2.2.3. Plasma Biomarkers of Endothelial and Vascular Inflammation

Compared to WT mice, the plasma concentrations of endothelial inflammation markers like E-selectin, VCAM-1, and ICAM-1 were increased in both groups of LDLr^−/−^.Leiden mice but decreased in db/db mice (Figure 3J,L,M; E-selectin: db/db: *p* = 0.003; LDLr^−/−^.Leiden+HFD+C: *p* = 0.002; LDLr^−/−^.Leiden+HFD: *p* = 0.004; VCAM-1: all experimental groups: *p* < 0.001; VCAM-1: db/db: *p* = 0.003; LDLr^−/−^.Leiden+HFD+C: *p* < 0.001; LDLr^−/−^.Leiden+HFD: *p* < 0.001). The concentration of P-selectin, another endothelial function indicator, was increased in db/db mice and LDLr^−/−^.Leiden mice fed with HFD compared to WT mice, whereas its concentrations did not change significantly in HFD+C fed LDLr^−/−^.Leiden mice (Figure 3K: db/db: *p* = 0.017; LDLr^−/−^.Leiden+HFD: *p* < 0.001). However, markers of dysmetabolism and thrombosis, such as leptin and plasminogen activator inhibitor-1 (PAI-1), showed significantly elevated plasma concentrations in all the experimental groups compared to WT mice (Figure 3N,O: db/db: *p* < 0.001; LDLr^−/−^.Leiden+HFD+C: *p* < 0.001; LDLr^−/−^.Leiden+HFD: *p* < 0.001).

### 2.3. Changes in BBB Integrity

#### 2.3.1. DCE-MRI

**Figure 4:**
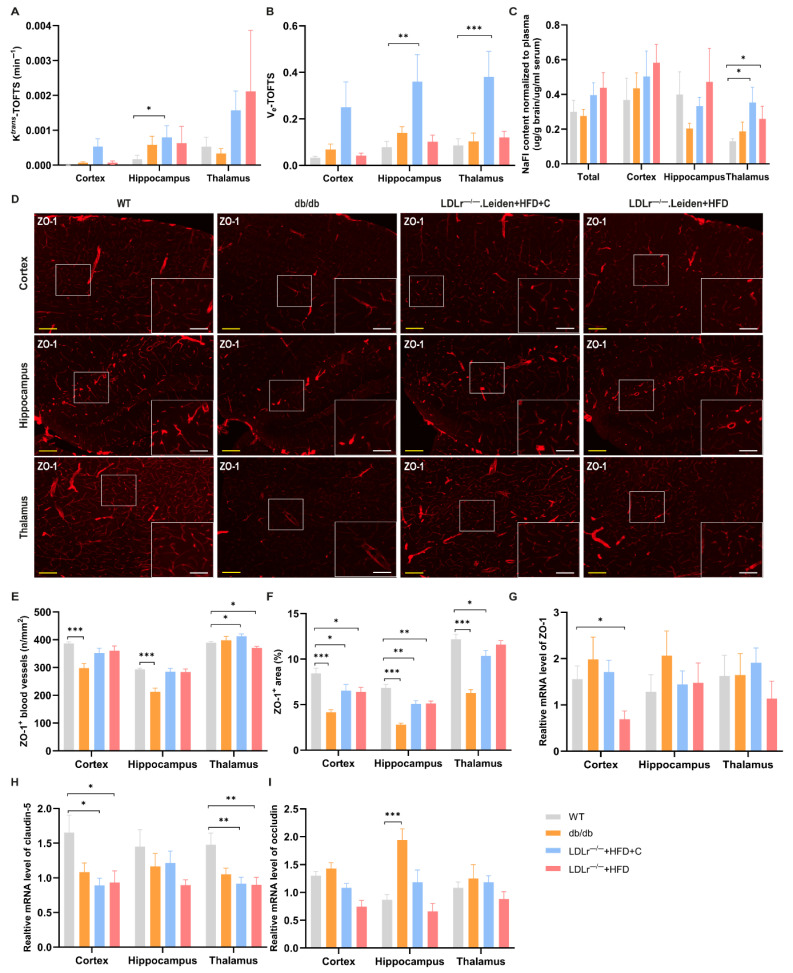
BBB integrity in different mouse models. (**A**) K^trans^ and (**B**) V_e_ of the TOFTS model are two important DCE-MRI parameters to detect BBB permeability (group size: WT: *n* = 7; db/db: *n* = 7; LDLr^−/−^.Leiden+HFD+C: *n* = 8; LDLr^−/−^.Leiden+HFD: *n* = 9). (**C**) The NaFl content normalized to plasma in total brain and different subregions (group size: WT: *n* = 10; db/db: *n* = 9; LDLr^−/−^.Leiden+HFD+C: *n* = 10; LDLr^−/−^.Leiden+HFD: *n* = 8). Immunohistochemical staining for zonula occludens-1 (ZO-1) was performed on the left hemisphere of the brain. (**D**) Representative images of ZO-1 staining in different ROIs (yellow scale bar = 300 µm, white scale bar = 100 µm). (**E**) The number of ZO-1 positive blood vessels per mm^2^ and (**F**) the percentage of ZO-1 positive staining per area were representative of ZO-1 positive vascular density (group size: WT: *n* = 11; db/db: *n* = 11; LDLr^−/−^.Leiden+HFD+C: *n* = 12; LDLr^−/−^.Leiden+HFD: *n* = 12). (**G**) ZO-1, (**H**) occludin, and (**I**) claudin-5 gene expression measured by qPCR (group size: WT: *n* = 11; db/db: *n* = 10; LDLr^−/−^.Leiden+HFD+C: *n* = 12; LDLr^−/−^.Leiden+HFD: *n* = 11). Data were presented as mean ± SEM. * *p* < 0.05, ** *p* < 0.01, *** *p* < 0.001.

The extended TOFTS model is a widely used pharmacokinetic model in DCE-MRI for evaluating BBB permeability [25]. K^trans^ is the most important BBB permeability-related DCE parameter, reflecting the transfer rate of gadobutrol from blood plasma into the tissue extravascular extracellular space (EES). In TOFTS models, LDLr^−/−^.Leiden mice fed a HFD+C showed a higher K^trans^ in the hippocampus compared to the WT group mice, indicating increased BBB leakage in this group of mice (Figure 4A, *p* = 0.028).

The parameter V_e_ refers to the fractional volume of the EES reflecting the space available within the tissue interstitium for accumulating gadolinium. In HFD+C-fed LDLr^−/−^.Leiden mice, V_e_ was significantly increased in both the hippocampus and the thalamus (Figure 4B, hippocampus: *p* = 0.003; thalamus: *p* < 0.001).

#### 2.3.2. Sodium Fluorescein (NaFl)

After NaFl injection via the tail vein, the NaFl content in brain tissue was used to investigate the BBB integrity, thereby supporting the results of DCE imaging with gadobutrol. The NaFl content in the thalamus of both groups of LDLr^−/−^.Leiden mice was significantly higher than that of WT mice (Figure 4C, LDLr^−/−^.Leiden+HFD+C: *p* = 0.044; LDLr^−/−^.Leiden+HFD: *p* = 0.036). No statistically significant differences were found in other subregions or between db/db mice and WT mice.

#### 2.3.3. TJs Integrity

Zonula occludens-1 (ZO-1), occludin and claudin-5 are the main constituent proteins of TJs, and their reduced expression and rearrangement impact the permeability of the BBB [26].

A lower amount of ZO-1 positive blood vessels was observed in the cortex and hippocampus of db/db mice compared to WT mice (Figure 4E, cortex: *p* < 0.001, hippocampus: *p* < 0.001). The density of ZO-1 positive blood vessels was also significantly reduced in the thalamus of LDLr^−/−^.Leiden mice fed with HFD (Figure 4E, *p* = 0.043). However, in contrast to the results of the other two groups of mice, the density of ZO-1 positive blood vessels in the thalamus of LDLr^−/−^.Leiden mice fed with HFD+C was significantly higher than that of WT mice (Figure 4E, *p* = 0.035). Consistent with the results of the ZO-1 positive vessels, smaller ZO-1 positive areas were observed in all subregions of all three groups, except for the thalamus of the LDLr^−/−^+HFD group (Figure 4F, db/db: cortex (*p* < 0.001), hippocampus (*p* < 0.001), thalamus (*p* < 0.001); LDLr^−/−^.Leiden+HFD+C: cortex (*p* = 0.048), hippocampus (*p* = 0.003), thalamus (*p* = 0.039); LDLr^−/−^.Leiden+HFD: cortex (*p* = 0.015), hippocampus (*p* = 0.002)).

We next examined the mRNA expression of the three TJ markers. The ZO-1 gene expression was decreased only in the cortex of LDLr^−/−^.Leiden+HFD mice compared to WT mice (Figure 4G, *p* = 0.014). Similar lower gene expression was observed in the cortex (Figure 4H, LDLr^−/−^.Leiden+HFD+C: *p* = 0.017; LDLr^−/−^.Leiden+HFD: *p* = 0.029) and thalamus (Figure 4H, LDLr^−/−^.Leiden+HFD+C: *p* = 0.007; LDLr^−/−^.Leiden+HFD: *p* = 0.009) of both LDLr^−/−^.Leiden groups when considering the claudin-5 gene expression. Conversely, qPCR results showed an increased occludin gene expression in the hippocampus of db/db mice (Figure 4I, *p* < 0.001).

### 2.4. Neuroinflammation

#### 2.4.1. Ionized Calcium-Binding Adapter Molecule 1 (IBA-1)

**Figure 5:**
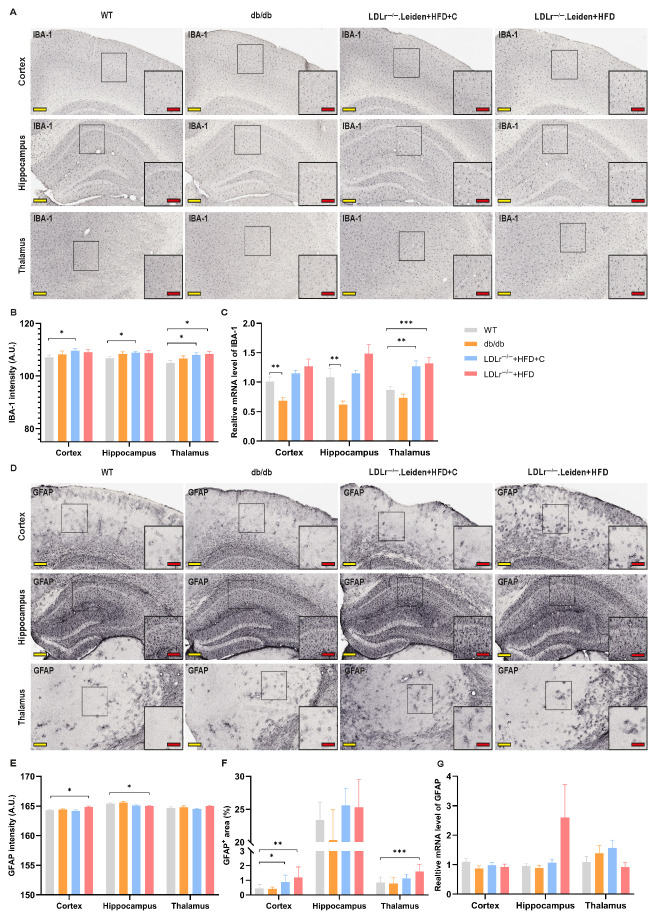
Neuroinflammation changes in different groups. Immunohistochemical staining for IBA-1 and glial fibrillary acidic protein (GFAP) was performed on the left hemisphere of the brain. (**A**) Representative images of IBA-1 immunohistochemistry staining in different ROIs (yellow scale bar = 400 µm; red scale bar = 100 µm). (**B**) The intensity of IBA-1 represented the amount of IBA-1 (group size: WT: *n* = 11; db/db: *n* = 11; LDLr^−/−^.Leiden+HFD+C: *n* = 12; LDLr^−/−^.Leiden+HFD: *n* = 12). (**C**) IBA-1 gene expression measured by qPCR (group size: WT: *n* = 11; db/db: *n* = 10; LDLr^−/−^.Leiden+HFD+C: *n* = 12; LDLr^−/−^.Leiden+HFD: *n* = 11). (**D**) Representative images of GFAP immunohistochemistry staining in different ROIs (yellow scale bar = 400 µm; red scale bar = 100 µm). (**E**) The intensity of GFAP represented the amount of GFAP (group size: WT: *n* = 10; db/db: *n* = 11; LDLr^−/−^.Leiden+HFD+C: *n* = 11; LDLr^−/−^.Leiden+HFD: *n* = 12). (**F**) The percentage of GFAP-positive area in different ROIs represented the distribution of activated astrocytes (group size: WT: *n* = 10; db/db: *n* = 11; LDLr^−/−^.Leiden+HFD+C: *n* = 11; LDLr^−/−^.Leiden+HFD: *n* = 12). (**G**) GFAP gene expression measured by qPCR (group size: WT: *n* = 11; db/db: *n* = 10; LDLr^−/−^.Leiden+HFD+C: *n* = 12; LDLr^−/−^.Leiden+HFD: *n* = 11). Data were presented as mean ± SEM. * *p* < 0.05, ** *p* < 0.01, *** *p* < 0.001.

When compared to WT mice, a higher intensity of IBA-1 was found in all brain subregions of LDLr^−/−^.Leiden mice fed with HFD+C (Figure 5B, cortex: *p* = 0.038; hippocampus: *p* = 0.01; thalamus: *p* = 0.019) and in the thalamus of LDLr^−/−^.Leiden mice fed with HFD (Figure 5B, *p* = 0.017). QPCR results showed lower IBA-1 gene expression in the cortex and hippocampus of db/db mice (Figure 5C, cortex: *p* = 0.003, hippocampus: *p* = 0.003), but higher expression in the thalamus of both LDLr^−/−^ groups of mice when compared with WT mice (Figure 5C, LDLr^−/−^.Leiden+HFD+C: *p* = 0.001; LDLr^−/−^.Leiden+HFD: *p* < 0.001).

#### 2.4.2. Glial Fibrillary Acidic Protein (GFAP)

As shown in Figure 5D,E, the intensity of GFAP was higher in the cortex (*p* = 0.022) but lower in the hippocampus (*p* = 0.043) of LDLr^−/−^.Leiden+HFD mice compared to WT mice. However, the relative GFAP-positive area was larger in the cortex (Figure 5F, *p* = 0.006) and thalamus (Figure 5F, *p* < 0.001) of LDLr^−/−^.Leiden+HFD mice. Similar to the HFD-fed LDLr^−/−^.Leiden mice, a larger GFAP-positive area was also seen in the cortex of LDLr^−/−^ mice fed with HFD+C (Figure 5F, *p* = 0.028). No significant changes in the GFAP gene expression were observed in both LDLr^−/−^.Leiden groups compared to WT controls (Figure 5G).

## 3. Discussion

The purpose of our study was to characterize and validate a translational mouse model for cSVD, in which BBB pathology, endothelial and vascular dysfunction and neuroinflammation can be studied. Such a model should display phenotypes and cSVD risk factors that recapitulate the disease in humans, including hypertension and hypercholesterolemia. Our results revealed that LDLr^−/−^.Leiden mice fed a HFD and HFD+C exhibited cSVD characteristics, including reduced CBF, brain atrophy, impaired endothelial and BBB function, as well as vascular inflammation and neuroinflammation. While db/db mice also showed some signs of TJs and endothelial dysfunction based on decreased ZO-1 expression and lower GLUT-1 transporters, no signs of BBB leakage were detected via DCE-MRI or NaFl analysis, and no vascular or neuroinflammation was observed.

We evaluated two mouse strains, db/db and LDLr^−/−^.Leiden mice, for their relevance in investigating BBB pathology in cSVD. Among them, LDLr^−/−^.Leiden mice, particularly those subjected to long-term HFD, emerged as more suitable for studying cSVD. Consistent with previous reports [27,28], these mice developed key cSVD risk factors, including obesity, hyperinsulinemia, and human-like dyslipidemia characterized by increased VLDL and LDL lipoprotein particles. Additionally, these mice have been shown to exhibit other cSVD risk factors like hypertension and microvascular impairments [22]. In our previous study, midlife LDLr^−/−^.Leiden mice on long-term HFD successfully replicated hallmarks of cSVD features, such as WMH, neuroinflammation and cognitive dysfunction observed in humans [29]. These mice also developed midlife obesity comparable to that seen in humans [29], reinforcing the model’s translational relevance. The HFD+C-fed LDLr^−/−^.Leiden mice displayed overweight, hypertriglyceridemia and hypercholesterolemia. However, their obesity was mainly manifested as perigonadal obesity, which was different from the mesenteric obesity induced by HFD alone [20]. Prolonged exposure to HFD+C may induce advanced fibrosis and severe atherosclerosis, which increase the mortality risk, thereby limiting its suitability for studies focusing on midlife obesity and reducing its translational relevance [20,21,30]. db/db mice developed spontaneous obesity and hyperinsulinemia as documented in the studies [17,18]. However, they suffer from high mortality around 36 weeks of age due to severe metabolic decompensation and diabetes-related complications, which limit their use in long-term studies. Thus, db/db mice serve as a model for diabetes-related cSVD, but their limited lifespan constrains their applicability for prolonged investigations. Altogether, neither db/db mice nor HFD+C fed LDLr^−/−^.Leiden mice are ideal for investigating cSVD mechanisms that mimic midlife situations. LDLr^−/−^.Leiden mice under prolonged HFD better recapitulate midlife obesity-associated cSVD, exhibiting key pathological features and broader translational relevance for BBB-focused research.

Cerebral hypoperfusion caused by changes in cerebrovascular structure and function is often expressed by impaired vasoreactivity and has been associated with the severity of cSVD [1,31,32]. Our results showed that hippocampal CBF in db/db mice and HFD-fed LDLr^−/−^.Leiden mice were lower than those of WT mice under conditions of both vasodilation and vasoconstriction, indicating impaired cerebrovascular function [33]. Notably, cortical CBF in HFD+C-fed LDLr^−/−^.Leiden mice was significantly lower than that of WT mice under vasodilation, with no difference observed under vasoconstriction. This contrasts with the behavior of healthy vessels, which normally regulate CBF by contracting and dilating in response to stimuli to maintain optimal oxygen supply in the brain [34]. This opposite behavior suggests impaired cortical vascular reactivity in HFD+C-fed LDLr^−/−^.Leiden mice, which aligns with current clinical findings of cSVD [35] and is potentially driven by endothelial dysfunction [34]. Moreover, impaired cerebral perfusion increases the risk of brain atrophy [36], one of the neuroimaging features of SVD, and can be evaluated by cortical thickness, brain and hippocampal volume. The presence of a thinner cortex in db/db mice and HFD-fed LDLr^−/−^.Leiden mice, as well as smaller brain and hippocampal volumes in db/db mice, suggest that both groups of mice show brain atrophy. These observations indicate the presence of cSVD in all experimental groups of mice.

Studies have found that endothelial dysfunction is reflected by decreased GLUT-1 expression and is associated with cognitive impairment [24]. In line with these prior studies, we found reduced GLUT-1 blood vessels and a smaller GLUT-1 positive area in LDLr^−/−^.Leiden mice, especially in the HFD+C fed LDLr^−/−^.Leiden group, demonstrating that both groups of LDLr^−/−^.Leiden mice had endothelial dysfunction [37]. Elevated plasma concentrations of endothelial inflammation indicators like E-selectin, P-selectin, VCAM-1 and ICAM-1 in LDLr^−/−^.Leiden mice further support the presence of endothelial dysfunction [38]. The increased PAI-1 and leptin concentrations implied a dysmetabolic proinflammatory state [39], possibly driven by endothelial dysfunction, further reinforcing the GLUT-1 findings. Additionally, high expression of these vascular inflammatory factors was also found in clinical trials, which are consistent with our results [40]. Given that glucose is the main energy substrate for the brain, the regionally increased GLUT-1 transporter expression in both groups of LDLr^−/−^.Leiden mice (in thalamus) and the increased hippocampal GLUT-1 mRNA expression in HFD-fed LDLr^−/−^.Leiden mice may represent a compensatory response to the reduced number of healthy capillaries to maintain the basic brain function [41]. Of note, db/db mice exhibited obvious regional variations in GLUT-1 expression, with more GLUT-1 positive vessels and less GLUT-1 expression per vessel in the cortex and hippocampus, whereas the opposite pattern was found in the thalamus. In addition, plasma concentrations of E-selectin, VCAM-1 and ICAM-1 were lower in db/db mice than in WT mice. We hypothesize that these conflicting results may result from altered blood glucose levels and cerebral perfusion of db/db mice [42,43], or represent compensatory mechanisms for endothelial dysfunction [41]. However, the specific regulatory mechanisms underlying these observations require further investigation. Taken together, these findings suggest that, compared to LDLr^−/−^.Leiden mice, db/db mice are less optimal for endothelial dysfunction in cSVD, due to their inconsistent endothelial marker profiles and compensatory GLUT-1 responses.

ECs are major components of the BBB, and their dysfunction is closely associated with BBB disruption. Our study used DCE-MRI to detect BBB permeability in mice, which has been the preferred imaging technique for assessing BBB failure in cSVD and other permeability applications in clinical practice [14,44]. The results of DCE-MRI revealed higher hippocampal K^trans^ and enlarged hippocampal and thalamic V_e_ in the group of HFD+C fed LDLr^−/−^.Leiden mice, revealing the presence of BBB leakage in this mouse model [45]. To our knowledge, this is the first application of DCE-MRI at such a high magnetic field strength (11.7 T) to detect BBB permeability in mice, and our findings are in agreement with clinical DCE-MRI observations [44]. These results were further validated by NaFl measurements, a typical BBB integrity detection method, which confirmed BBB leakage in the thalamus of both groups of LDLr^−/−^.Leiden mice. It is noteworthy that db/db mice did not show any BBB leakage by either DCE-MRI or NaFl, which contradicts other research results [46,47]. This discrepancy may stem from differences in experimental protocols, such as shorter post-injection intervals and anesthesia during NaFl assessment, which could limit the sensitivity of our approach, potentially underestimating subtle BBB changes in db/db mice. The lack of significant changes in K_ep_ in DCE-MRI results also supports the above hypothesis.

BBB dysfunction is also characterized by the loss of TJ components [26]. In our study, IHC revealed reduced ZO-1 expression across all experimental groups, indicating TJ compromise in both db/db and LDLr^−/−^.Leiden mice. These findings were in accordance with previous studies reporting TJ alterations in association with BBB impairment [48,49] and partially in line with our DCE-MRI and NaFl measurement findings, particularly in LDLr^−/−^.Leiden mice. Reduced cortical ZO-1 mRNA expression in HFD-fed LDLr^−/−^.Leiden mice, along with decreased cortical and thalamic claudin-5 mRNA expression across both diet groups, further substantiated the observed TJ damage. These findings confirmed the presence of BBB dysfunction in LDLr^−/−^.Leiden mice at both structural and functional levels [37]. In db/db mice, contradictory results were found where ZO-1 expression was downregulated and occludin expression was upregulated. These results were consistent with the conflicting GLUT-1 expression in db/db mice, which may provide more evidence for the hypothesis of a compensation mechanism. Although permeability assays did not detect overt BBB leakage in db/db mice, the TJ alterations suggest that subtle barrier dysfunction may already occur at a molecular or ultrastructural level. Nevertheless, the extent of BBB leakage in db/db mice was limited when compared to that in LDLr^−/−^.Leiden mice. In LDLr^−/−^.Leiden mice, BBB leakage detected by DCE-MRI and NaFl was predominantly observed in the thalamus, whereas TJ alterations were detected in both the cortex and thalamus. This discrepancy may indicate that structural TJ damage may precede measurable functional leakage. Overall, LDLr^−/−^.Leiden mice offer a more robust mouse model for studying BBB disruption than db/db mice.

BBB dysfunction facilitates the entry of peripheral harmful substances and immune cells into brain tissue, which in turn activates microglia and triggers an amplified neuroinflammation response [14,50]. Aligned with this, we found microglial activation in both LDLr^−/−^.Leiden mouse groups, represented by elevated IBA-1 expression. Moreover, astrocytes, as integral components of the BBB, can also respond to microglial activation and interact with other cells such as oligodendrocytes and neurons, thereby aggravating neuroinflammation [51]. In our study, astrogliosis was observed in both groups of LDLr^−/−^.Leiden mice, indicated by higher cortical and thalamic GFAP expression, which may reflect a reaction to BBB damage-induced microglial activation. Moreover, inflammation factors secreted by microglia and astrocytes can further compromise BBB integrity by disrupting endothelial function and TJ integrity [52], creating a vicious cycle of inflammation and BBB dysfunction. Notably, compared to WT mice, db/db mice showed less microglial activation, which contradicts findings from other studies showing increased neuroinflammation [53]. These differences could be attributed to the different leakage sites and various inflammatory response stages of different subregions [54]. Together, these observations further support our findings that both groups of LDLr^−/−^.Leiden mice exhibited endothelial dysfunction and BBB leakage, accompanied by neuroinflammatory responses, thus reinforcing their translational relevance as a model for cSVD-related BBB pathology.

This study has several limitations. First, the use of only male mice in this study represents a sex bias that limits the generalizability of our findings and overlooks well-documented sex-related differences in vascular function and cSVD pathology. In this early-stage model validation, the choice of male mice was made to reduce variability associated with hormonal cycles and to ensure consistency in experimental design. However, we recognize that sex differences can significantly affect cerebrovascular pathophysiology. Future studies will include female and age-matched cohorts to enable a more comprehensive assessment of sex-related effects in cSVD. Second, although blood glucose was not measured, the observed hyperinsulinemia in LDLr^−/−^.Leiden mice is consistent with our previous reports and is frequently accompanied by hyperglycemia, which likely reflects early insulin resistance [55,56]. This can occur prior to overt hyperglycemia and contributes to vascular and inflammatory changes [57]. Finally, we assessed most of the BBB-related markers using IHC and RT-PCR. While these techniques offer insight into spatial expression patterns and transcriptional changes, they do not fully capture dynamic alterations in protein abundance or structural integrity. Future studies should incorporate protein-level confirmation to strengthen the mechanistic interpretation of BBB dysfunction.

Moreover, the DCE-MRI and NaFl methods used to investigate BBB integrity yielded regionally inconsistent results, which may be attributed to several factors. First, vascular distribution varies significantly across brain regions, influencing tracer distribution and signal intensity. Prior evidence showed stronger contrast of NaFl in regions of dense blood vessels [58]. This is likely due to NaFl accumulation in damaged BBB areas through passive diffusion. The hippocampus, with its relatively sparse vascular network compared to the thalamus, for example, may result in limited NaFl accumulation and therewith reduced signal intensity. This may partly explain why subtle BBB leakage in the hippocampus is less evident in NaFl, while potentially being more detectable with dynamic methods like DCE-MRI. Second, the two methods differ in their detection time windows and data acquisition strategies. DCE-MRI enables real-time, dynamic monitoring of BBB permeability by continuously tracking the distribution of gadobutrol throughout the imaging period [59]. However, as NaFl examined postmortem, it reflects only endpoint accumulation of the dye [60]. Thus, NaFl may not detect transient or subtle BBB changes. Third, differences in the molecular size of contrast agents (gadobutrol: 604.73 Da, NaFl: 376.27 Da) may also influence detection sensitivity. Although gadobutrol is larger, its use in DCE-MRI benefits from kinetic modeling and dynamic acquisition, enabling the detection of subtle changes in BBB permeability that may not be observable with NaFl. In summary, while DCE-MRI offers dynamic and real-time in vivo evaluation of changes in BBB permeability, its quantification is more technically complex and susceptible to perfusion fluctuations and spatial resolution. NaFl, though limited to postmortem endpoint assessment, is more straightforward for detecting established leakage. Despite their inherent limitations, the complementary nature of DCE-MRI and NaFl provides a more comprehensive assessment of BBB integrity, highlighting the value of multimodal approaches in studying BBB function.

Summarizing, we successfully established and validated a mouse model for studying BBB changes in cSVD by comparing the different manifestations of db/db mice and LDLr^−/−^.Leiden mice. Among these, HFD-fed LDLr^−/−^.Leiden mice demonstrated superior suitability for clinical translation, as they exhibited notable endothelial and BBB dysfunction alongside physiological characteristics and risk factors associated with cSVD. Although similar pathological features were observed in HFD+C fed LDLr^−/−^.Leiden mice, the rapid cholesterol elevation over a short period in this group may limit their clinical translational potential and relevance for long-term translational studies. Nevertheless, this variant remains useful for investigating pathologies linked to severe hypercholesterolemia. As for db/db mice, although they also present several risk factors and evidence of endothelial dysfunction, they did not develop a larger spectrum of cSVD features with particularly BBB leakage and brain inflammation when compared to LDLr^−/−^.Leiden mice.

In conclusion, LDLr^−/−^.Leiden mice, particularly those maintained on a HFD, rather than db/db mice, offer a promising experimental model for exploring BBB disruption in the context of cSVD. While not fully replicating the complexity of human cSVD, this model captures key vascular features and pathological mechanisms relevant to disease progression. Importantly, building upon our previous findings that LDLr^−/−^.Leiden mice develop WMH and cognitive deficits resembling obesity-related cSVD in humans [29], this model appears to offer greater translational relevance than db/db mice. Notably, this is the first mouse model in which BBB damage has been demonstrated with multimodal translational DCE-MRI and postmortem biochemistry and IHC. Using this model, we can gain a comprehensive understanding of the BBB pathology in cSVD and study the sequence and potential mechanisms at the earliest stages of the disease. Furthermore, the model could also play an important role in identifying and testing potential therapeutic interventions.

## 4. Materials and Methods

### 4.1. Animals

For this study, 48 male mice were used. Mice were divided into four groups (12 mice per group) according to different strains and diets. Group 1 (WT group): C57BL/6J (WT) mice on a chow diet between 12 and 16 weeks of age; Group 2 (db/db group): db/db mice on a chow diet between 12 and 16 weeks of age; Group 3 (LDLr^−/−^.Leiden+HFD+C): LDLr^−/−^.Leiden mice on HFD+ 1% cholesterol between 12 and 16 weeks of age; Group 4 (LDLr^−/−^.Leiden+HFD): LDLr^−/−^.Leiden mice on HFD between 24 and 36 weeks of age see Figure 1. The C57/BL6J mice and db/db mice were obtained from Charles River Laboratory (Germany and Italy, respectively), and the LDLr^−/−^. Leiden mice were obtained from a specific pathogen-free breeding stock at TNO Metabolic Health Research (Leiden, The Netherlands). Throughout the entire experiment, mice were housed under standard housing conditions (21 °C temperature, 50–60% humidity, 12 h dark/light cycle) in digital ventilated cages (DVC; Tecniplast SPA, Buguggiate, VA, Italy) at the Preclinical Imaging Center (PRIME) of the Animal Research Facility (Radboudumc, Nijmegen, The Netherlands). The DVCs of the different groups were randomly placed on the racks in the animal room to ensure that all cages were under the same housing conditions. Home-cage activity was measured 24 h per day in the DVC, as described previously [61,62]. All mice were allowed free access to water and food. Mice were weighed at arrival and before MRI scanning.

All animal protocols used in this study were ethically approved by the TNO Animal Welfare Body and the Veterinary Authority of the Radboud University Medical Center (Nijmegen, The Netherlands; approval number: 2023-0011-001). All mouse experiments were performed and reported according to the Dutch federal law for animal experimentation and the ARRIVE guidelines. MRI was performed during the daytime between 7:00 a.m. and 6:00 p.m. The study design is detailed in Figure 1.

### 4.2. Home-Cage Activity: Digital Ventilated Cages (DVC)

Animals were group housed in DVCs (Tecniplast S.p.A., Buguggiate, VA, Italy) with 2 or 3 mice per cage during the experiment to record mouse activity 24/7 [63]. More specifically, Animal Locomotion Index (ALI) during day and nighttime was captured and measured automatically via the DVC metric system. All explanations of system-specific details were previously described [62,64]. We selected DVC data from the last 3 days before MRI scanning. Relative activity per mouse was expressed as the average of measured ALI divided by the number of mice per cage.

### 4.3. Brain MRI

After a two-week acclimatization, MRI was performed using an 11.7 T BioSpec Avance III small animal MR system (Bruker Biospin, Ettlingen, Germany) with 600 mT/m actively shielded gradients and Paravision 6.0.1 software (Bruker, Karlsruhe, Germany), as described previously [61,65,66]. Mice were fully anesthetized and maintained under inhaled isoflurane anesthesia (induction: 3.5%, maintenance: ~1.5%; Abbott Animal Health, Abbot Park, IL, USA) in a medical air and oxygen mixture (2:1). Under anesthesia, mice were prepared with intravenous tail vein catheters for subsequent contrast agent injection. Mouse heads were immobilized during scanning in a stereotactic device to minimize movement artifacts. Breathing rate was monitored using a pneumatic cushion respiratory monitoring system (Small Animal Instruments Inc., Stony Brook, New York, NY, USA) and body temperature was monitored with a fiber optic rectal thermometer and maintained at 37 °C with the help of a water heating pad. A circular plastic phantom filled with contrast agent was placed on the head of the mouse as a reference.

#### 4.3.1. Dynamic Contrast-Enhanced Magnetic Resonance Imaging (DCE-MRI)

DCE-MRI was used to evaluate the dynamic distribution of contrast agent in tissues and the changes in signal intensity to assess the hemodynamic characteristics. Gadobutrol (molecular size = 604.73 g/mol) serves as a contrast agent, which could amplify the signal, and is thought not to cross an intact BBB [67].

For DCE-MRI acquisition, T1 mapping was performed based on fitting measurements with varying TR (5500 ms, 3000 ms, 1500 ms, 800 ms, and 400 ms) and TE (200 ms) using ROCKETSHIP [68,69]. A two-dimensional T1-weighted FLASH sequence with a matrix of 256 × 256 pixels was used. The FOV (25 × 25 × 1 mm^3^) was positioned to cover both the circular plastic filling with contrast agent and the brain at bregma −1.94. Spatial resolution was 0.098 × 0.098 × 1 mm^3^, temporal resolution of 1919 ms, 480 repetitions, with a total length of 15 min and 21 s. Anti-aliasing in Z was 2.0, flip angle = 15°, TR = 10.0 ms, and TE = 1.72 ms.

After T1 mapping scanning, contrast injection was initiated, consisting of an 80 µL saline bolus + different volumes of 70 mM gadobutrol (Gadovist, Bayer, Germany) in saline, followed by a 45 µL saline bolus. All fluid injections were performed manually by an experienced technician at a consistent speed and duration. Due to the significant differences in body weight among different groups of mice, and considering that the total blood volume of mice varies relatively little with body weight, we calculated the required volume of contrast agent based on the blood volume of the mice to ensure a similar concentration of contrast agent in the blood across different groups of mice. We estimated the approximate range of the blood volume of the mice based on previous literature [70].

The pre-gadobutrol and post-gadobutrol images were collected for analysis. Voxel-wise pharmacokinetic modeling was performed with the software ROCKETSHIP version 1.2 (https://github.com/petmri/ROCKETSHIP (accessed on 6 December 2023) [68]. The extended TOFTS model and Patlak model are widely used in DCE-MRI for quantifying tissue perfusion and permeability. Permeability parameters like contrast agent efflux transfer constant (K^trans^), contrast agent reflux transfer constant (K_ep_), and the extravascular extracellular space (EES) volume fraction (V_e_) were derived from DCE-MRI based on the above model [71]. Reference regions were drawn by two experienced researchers in the bilateral temporal muscles seen at bregma −1.94 [68]. Global drift correction was performed using the circular plastic filling with gadobutrol as drift ROIs. For calculation, the needed T1 relaxivity of the contrast agent (1/s*mM) was 4.02, calculated as previously reported [72,73]. Both models showed the same effects. We focused on TOFTS as this approach has advanced permeability parameters. 

#### 4.3.2. Cortical Thickness and Volumetry

Two-dimensional T2-weighted turbo rapid imaging with refocused images covering the entire mouse brain was acquired in three directions. Intracranial volume was measured manually with ITK-SNAP (bregma 4.28 to −5.88) [74]. The ventricular volume was directly calculated with the FMRIB Software Library (version 5.0.10) using the threshold-based method. Brain volume was calculated by subtracting ventricular volume from intracranial volume. Cortical thickness and hippocampal volume were measured manually with ImageJ (v1.53, National Institutes of Health, Bethesda, MD, USA) on coronal images (matrix = 256 × 256 pixels, 34 slices, slice thickness = 0.5 mm, FOV = 20 × 20 mm, spatial resolution = 0.078 mm, flip angle = 90°, TE = 30 ms, TR = 3226.6 ms, RARE factor = 8, and averages = 3). The ROIs were selected based on the mouse brain atlas of Paxinos and Franklin [74]. The cortical thickness was measured at four different Bregma levels: motor cortex (bregma 1.10), somatosensory cortex (bregma −0.94), auditory cortex (bregma −2.46), and visual cortex (bregma −2.46) in both right and left hemispheres. For each region, the measures from the left and right hemispheres were averaged. The total cortical thickness was calculated as the average of all measurements. Hippocampal volumes were manually measured on 6 consecutive slices to cover the entire hippocampus (bregma −1.34 to −3.40). After segmentation, the hippocampal volume was calculated using the following formula: the hippocampal volume = sum of ROI × slice thickness (0.5 mm).

#### 4.3.3. Arterial Spin Labeling (ASL)

CBF was measured using an ASL sequence with the flow-sensitive alternating inversion recovery (FAIR) method in different ROIs (cortex, hippocampus, and thalamus) [75]. The regional CBF was measured using the same protocol as described previously [61]. Briefly, mice were exposed under a normal gas mix (medical air:oxygen = 2:1) to measure the CBF under vasodilative conditions. Subsequently, mice were exposed under pure oxygen (medical air:oxygen = 0:3) to measure the CBF under vasoconstrictive conditions. 

### 4.4. Perfusion and Tissue Preparation

After the MRI, sodium fluorescein (NaFl, Sigma-Aldrich, St. Louis, MO, USA, 25 µg/g body weight, molecular size = 376.27 g/mol) was injected via the still-present intravenous cannula after the MRI scanning, and the mice were still under anesthesia. Ten minutes after NaFl injection, mice were transcardially punctured to collect blood samples and thereafter sacrificed by transcardial perfusion with 0.1 M phosphate-buffered saline (PBS) at room temperature. The blood samples were centrifuged at 2000 rpm at 4 °C for 10 min, and the supernatant was collected. Finally, these tubes were snap frozen in liquid nitrogen and stored at −80 °C.

The brains were removed from the skull and separated into the left and right hemispheres. Left hemispheres of each brain were collected and subsequently fixed in 4% paraformaldehyde for 24 h. Then the tissue was transferred to 0.1 M PBS containing 0.01% sodium azide and stored at 4 °C for postmortem immunohistochemical studies. The right hemispheres were subdivided into cortex, hippocampus and thalamus. The brain subregions were weighed and rapidly frozen in liquid nitrogen and thereafter stored at −80 °C for NaFl measurements.

### 4.5. Blood Chemistry

Plasma total cholesterol and triglyceride (TG) concentrations were assayed in EDTA-plasma with commercially available enzymatic assays (GPO-PAP and CHOD-POP, respectively, Roche Diagnostics, Almere, The Netherlands). The concentrations of plasma biomarkers were analyzed using ELISA assays from R&D Systems (Abingdon, UK): VCAM-1 (kit DY643), ICAM-1 (kit DY796), E-selectin (kit DY575), P-selectin (kit DY737), plasminogen activator inhibitor-1 (PAI-1) (kit DY3828), and leptin (kit DY498). Plasma concentrations of ApoB were determined with an assay (ab230932) from Abcam (Waltham, MA, USA). For lipoprotein profiles, pooled plasma for each group of mice was fractionated using an AKTA fast protein liquid chromatography system (Pharmacia, Roossendaal, The Netherlands). Fractions were collected, and cholesterol and triglycerides were analyzed in each fraction. Plasma insulin was determined by ELISA (Chrystal Chem Inc., Downers Grove, IL, USA).

### 4.6. NaFl Measurement

We optimized our protocol based on previous literature [76]. Briefly, after thawing on ice, brain tissues (cortex, hippocampus and thalamus from right hemispheres) were fully ground in PBS (250 µL for cortex and hippocampus, 500 µL for thalamus). A total of 15 µL plasma was diluted in 150 µL PBS. Next, the samples were centrifuged at 14,000× *g* for 10 min at 4 °C. The supernatant was transferred into new Eppendorf tubes, and the pellets were stored for subsequent testing. An equal volume of ethanol (98%) was added into the tubes. After mixing by swiveling, the mixture was incubated on ice for 30 min. Subsequently, the tubes were centrifuged for 10 min at 14,000× *g*, followed by the collection and measurement of the supernatant. Finally, 100 µL of supernatant from each sample was used for NaFl measurement.

The fluorescence was determined (excitation at 490 nm and emission at 530 nm) using a microplate reader (SpectraMax iD5, Molecular Devices, San Jose, CA, USA). Calculations were based on external standards in the same solvent (ranging from 1 to 250 ng/mL). The tissue content was quantified from linear standard curves derived for each of the dyes and expressed per gram of tissue. The final values were normalized to serum NaFl.

### 4.7. Real-Time Quantitative PCR (RT-qPCR)

RNA was extracted from the pellets from procedure 4.2 using the TRIzol method described previously [77]. cDNAs were synthesized using RNase-free DNase I (RQ1, Promega, Fitchburg, MA, USA) and Maxima H Minus cDNA Synthesis Master Mix (Thermo Fisher Scientific, Waltham, MA, USA). Subsequently, quantitative PCR was exerted in 300-well plates (Thermo Fisher Scientific) using SYBR Green master mix (Thermo Fisher Scientific, Waltham, MA, USA) on a StepOnePlus system (Thermo Fisher Scientific, Waltham, MA, USA). Glyceraldehyde-3-phosphate dehydrogenase (GAPDH) and hypoxanthine guanine phosphoribosyl transferase (HPRT) were used as the reference genes for normalization. The primer sequences are listed in Table 1.

### 4.8. IHC

The fixated left hemispheres were sectioned into 30 μm free-floating coronal sections using a sliding microtome (Microm HC 440, Walldorf, Germany). A total of eight series were obtained, and complete series were used for each staining.

#### 4.8.1. IBA-1, GFAP and GLUT-1 Staining

Ionized calcium-binding adapter molecule 1 (IBA-1) and glial fibrillary acidic protein (GFAP) were used to assess, respectively, activated microglia and astrocytes reflecting neuroinflammation [50,51]. Glucose transporter-1 (GLUT-1) staining was performed for measuring microvascular and endothelial function [24]. Free-floating frozen sections were stained following previously described standard immunohistochemical procedures [77]. Sections were separately incubated with polyclonal goat anti-IBA-1 antibody (1:6000, Abcam, Cambridge, UK), polyclonal rabbit anti-GFAP antibody (1:80,000, Agilent Technologies, Santa, Clara, CA, USA) or polyclonal rabbit anti-GLUT-1 antibody (1:40,000, Millipore, MA, USA). Donkey anti-goat biotin (1:1500, Jackson Immunoresearch, Cambridgeshire, UK), respectively. Donkey anti-rabbit biotin (1:1500, Thermo Fisher Scientific, Waltham, MA, USA) was used as a secondary antibody.

#### 4.8.2. ZO-1 Staining

To investigate the BBB integrity, immunofluorescence staining on the TJs component zonula occludens 1 (ZO-1) was performed. Brain sections were rinsed three times with PBS and preincubated for 30 min in blocking solution (3% BSA and 0.5% Triton X-100 in PBS). After rinsing three times with PBS, sections were incubated with monoclonal rabbit anti-ZO-1 antibody (1:2000, Abcam, Cambridge, UK) for 3 h at room temperature in the dark. Then, sections were incubated with anti-rabbit-Alexa Fluor Plus 647 (1:2000, Thermo Fisher Scientific, Waltham, MA, USA) and 4′,6-diamidino-2-phenylindole (DAPI) (1:1000, Thermo Fisher Scientific, Waltham, MA, USA) following rinsing in PBS in the dark. Finally, after rinsing three times with PBS, sections were mounted on gelatin-coated slides and covered with coverslips and dried overnight.

#### 4.8.3. Quantification

All immunohistochemically stained sections were scanned at 20× magnification with a digital slide scanner (Aperio AT2, Leica Biosystems, Amsterdam, The Netherlands). Only for the ZO-1 staining, images were captured at 10× magnification using a Zeiss Axioscop microscope (Zeiss, Jena, Germany). Brain sections at bregma −1.94 were selected according to the Franklin and Paxinos mouse atlas and quantified by two researchers in a double-blind fashion [74]. The cortex (bregma −1.94), hippocampus (bregma −1.94) and thalamus (bregma −1.94) were manually selected by researchers, and the ROI area was chosen for further analysis when two researchers reached a consensus.

The intensity of GLUT-1, IBA-1 and GFAP staining, representing the amount of GLUT-1, IBA-1 and GFAP, was automatically measured by ImageJ and calculated by subtracting the mean particle intensity from background intensity. Moreover, the positive particle number per area and the relative positive area (stained area/manually drawn ROI) were counted and measured by ImageJ at 5× magnification.

The positive ZO-1 number per area and the relative ZO-1 positive area were measured in the same manner and regions as described above. Since the ZO-1 antibody is biotin-labeled, the ZO-1 intensity was directly detected automatically by ImageJ without the need for subsequent conversion.

### 4.9. Statistics

Data were analyzed using IBM SPSS Statistics 27 (IBM Corporation, Armonk, NY, USA). All data were examined for outliers (technical outliers and statistical outliers) and normality. Data transformation according to Tukey’s Ladder of Powers was performed if data were not normally distributed. Uni-resp. multivariate ANOVA with Bonferroni correction for multiple testing (e.g., number of ROI) was used to evaluate the differences among groups when the data were normally distributed or could be transformed to a normal distribution. The Kruskal–Wallis test was performed for non-normally distributed data. Statistical significance was considered by a *p*-value less than 0.05 (* *p* < 0.05, ** *p* < 0.01,*** *p* < 0.001). Data are expressed as mean ± standard error of the mean (SEM).

## Figures and Tables

**Table 1:** The sequences of primers.

Name	Forward	Reverse
Occludin	CCACCCCCATCTGACTATGC	TTCAGGCACCAGAGGTGTTG
Claudin-5	GTTAAGGCACGGGTAGCACT	TACTTCTGTGACACCGGCAC
ZO-1	CTCCGTTGCCCTCACAGTAC	ACTGAGTTGCCTTCACCCTG
GLUT-1	GATCCCAGCAGCAAGAAGGT	TAGCCGAACTGCAGTGATCC
IBA-1	GGATTTGCAGGGAGGAAAAG	TGGGATCATCGAGGAATTG
GFAP	TCGGCCAGTTACCAGGAGG	ATGGTGATGCGGTTTTCTTCG
HPRT	TGATTAGCGATGATGAACCAGGT	AGCAAGTCTTTCAGTCCTGTCC
GAPDH	GTCGGTGTGAACGGATTTGG	ACAATCTCCACTTTGCCACTG

## Data Availability

The datasets generated and/or analyzed during the current study are available upon reasonable request to the corresponding author.

## Abbreviations

The following abbreviations are used in this manuscript:

cSVD	cerebral small vessel disease
MRI	magnetic resonance imaging
WMH	white matter hyperintensities
CBF	cerebral blood flow
BBB	blood–brain barrier
ECs	endothelial cells
TJs	tight junctions
ICAM-1	intercellular adhesion molecule 1
VCAM-1	vascular cell adhesion molecule 1
HFD	high-fat diet
HFD+C	high-fat diet with 1% cholesterol
ASL	arterial spin labeling
DVC	digital ventilated cages
NaFl	sodium fluorescein
PBS	phosphate-buffered saline
EDTA	ethylenediaminetetraacetic acid
ELISA	enzyme-linked immunosorbent assay
TG	triglycerides
PAI-1	plasminogen activator inhibitor-1
GAPDH	glyceraldehyde-3-phosphate dehydrogenase
HPRT	hypoxanthine guanine phosphoribosyl transferase
IHC	immunohistochemistry
IBA-1	ionized calcium-binding adapter molecule 1
GFAP	glial fibrillary acidic protein
GLUT-1	glucose transporter-1
ZO-1	zonula occludens 1
DAPI	4′,6-diamidino-2-phenylindole
EES	extravascular extracellular space
